# Three-Dimensional Data Acquisition Methods and Their Use in River Levee Topographic Survey

**DOI:** 10.3390/s26030841

**Published:** 2026-01-27

**Authors:** Junko Kaneto, Satoshi Nishiyama, Keisuke Yoshida

**Affiliations:** Graduate School of Environmental, Life, Natural Science and Technology, Okayama University, Okayama 700-8530, Japan; nishiyama.satoshi@okayama-u.ac.jp (S.N.);

**Keywords:** mobile mapping system, external shape of levee, laser irradiation, high-precision survey

## Abstract

Frequent heavy rainfalls due to climate change in recent years have led to an increasing incidence of severe damage, such as levee breaches. However, the integrity of levees is currently assessed by visual inspection, relying on the skill and experience of the overseeing engineers. Future work requires close monitoring of the external shape of levees and the implementation of quantitative assessments if abnormalities such as deformation are discovered. Therefore, the mobile mapping system (MMS), which uses a vehicle-mounted laser scanner to conduct surveys while moving, has attracted attention as a method for conducting high-precision surveys. However, the presence of blind spots in the laser irradiation indicates that there is no practical method for identifying areas that require countermeasures for the entire levee. In this paper, we discuss the appropriate position of laser irradiation that allows data acquisition down to the toe of the slope, and then propose a method of laser irradiation from a high altitude. Compared to previous laser surveys using vehicles, this method was able to obtain a high-density laser point cloud over the entire levee, demonstrating that it is possible to detect detailed deformations not only on the crest of the levee but also on the slope.

## 1. Introduction

Global-scale climate change in recent years has led to an increase in extreme precipitation, consequently leading to increased damage from flooding. By the end of this century, average annual rainfall is predicted to increase by a factor of approximately 1.1–1.3, which will increase the importance of maintaining and managing river channels and river management facilities [1,2,3]. River levees are particularly long structures and are stipulated to be constructed with embankments, but a single breach could pose a risk of widespread damage. Current levees have undergone numerous modifications, including raising the levee height and widening, in order to improve the river channel’s flow capacity or to prevent erosion and scouring. However, there is a lack of information on the internal structure and geological features of embankments in many cases. Currently, river inspections are typically conducted visually, and assessment of levee integrity relies on the experience of engineers [4]. Responding to current climate change scenarios requires closely monitoring the external shape of levees during normal times, and taking prompt measures if deformation or other abnormalities are found.

Considering past cases, aspects that should be considered as signs that a levee may no longer be able to function include bulging and slope collapse, as well as other topographical or geological changes as listed [5,6].

**Figure 1:**
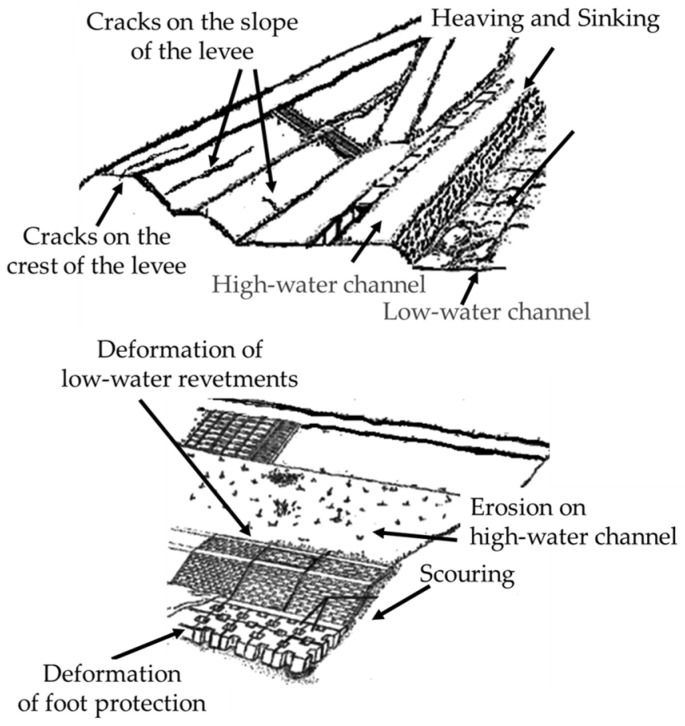
Examples of levee deformations. (**Top**) Deformations leading to levee collapse. (**Bottom**) Deformations leading to lateral erosion.

- Cracks on the crest or slope of a levee may indicate the formation of a sliding surface on the levee. If left unchecked, the levee will lose its shear resistance when the seepage surface inside the levee rises, resulting in crack expansion and sliding failure. Therefore, it is important not to overlook deformations that accompany crack expansion. The top part of Figure 1 is an example of deformation that can lead to levee failure.
- Deformation of the low-water revetment causes lateral erosion of the high-water bank because of progressive destruction due to fluid forces. Erosion of the floodplain also leads to the risk of the embankment itself becoming destroyed by erosion. Therefore, structural deformation that can lead to lateral erosion of the levee slopes, as shown in the bottom part of Figure 1, requires quantitative evaluation of the location, size, and progression of the deformation.

**Figure 2:**
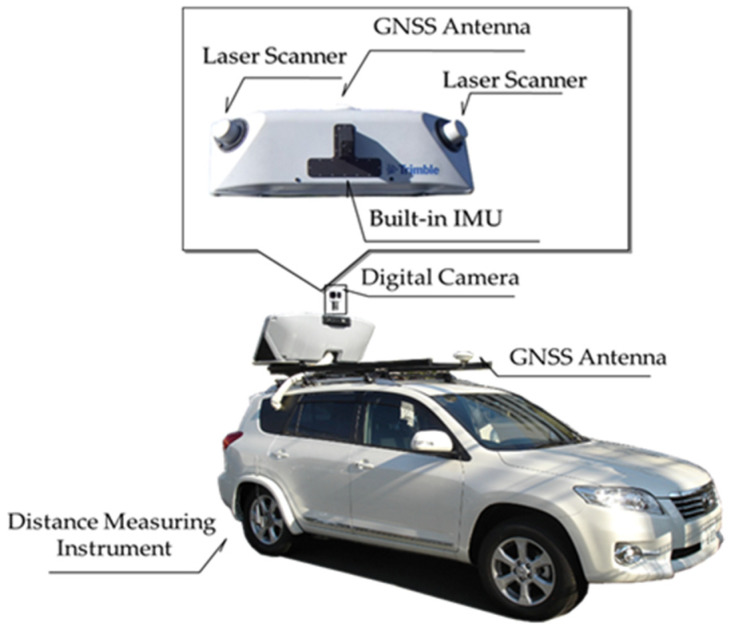
Example of configuration of MMS surveying system.

Furthermore, rainwater has been observed to infiltrate the levee slope from the levee crest, increasing pore water pressure within the slope, which decreases soil strength and results in slope collapse. It is recommended to add pavement on the levee crest to prevent levee infiltration; however, this can also increase the infiltration of rainfall from the levee crest to the slope, increasing the risk of slope erosion and the occurrence of loosened areas. In particular, levee slopes with soil covering tend to be more susceptible to soil strength deterioration due to the high hydraulic conductivity of the slope, which promotes rainfall infiltration. However, the soil structure inside the levee is often unknown and difficult to investigate, so detecting detailed changes in the longitudinal and cross-sectional shape of the levee is crucial to ensure the above-mentioned phenomena are identified [7,8]. The frequency of levee inspections does not need to be increased to detect such levee deformation. However, subtle changes in levee shape cannot be quantitatively detected by visual inspection. Thus, current inspection methods, which rely primarily on visual inspection, run the risk of overlooking these subtle changes. Surveys are conducted at intervals of, for example, 200 m, in order to observe changes in the shape of river levees. Yet, detecting subtle changes in the shape of the levee is impossible without three-dimensional data that captures continuous planar deformation. However, only limited personnel are available in reality for river levee inspections over long distances. Several cases have been reported in which 3D topographical surveys are conducted using laser scanners mounted on aircrafts, unmanned aerial vehicles (UAVs), or vehicles, and the data captured are used to quantitatively identify the locations of deformation [9,10,11,12,13]. In particular, mobile mapping systems (MMSs), which can continuously acquire laser point clouds while traveling, have attracted attention as a method for quickly conducting high-precision surveys [14,15,16]. As shown in Figure 2, the MMS is equipped with a digital camera and laser scanner on a vehicle. It calculates its own position using RTK GNSS kinematic analysis and correlates GNSS time with odometer readings and attitude information at the time of laser irradiation determined by an inertial measurement unit (IMU). This allows the MMS to measure 3D coordinates around the vehicle while it is moving [17,18].

Several studies using MMSs to survey river levees have been conducted. For example, a study examined the relationship between the accuracy of a vehicle’s own position while moving and surveying accuracy, and the results showed that surveying accuracy deteriorated as the baseline length increased. This highlights the importance of the accuracy of determining the vehicle’s position, that is, selecting self-positioning electronic reference points. Furthermore, another study used these results as a basis to demonstrate that highly accurate detection of levee deformation can be achieved by using a virtual reference point (VRS) method to reduce the baseline length to 5 km or less, and performing height correction using adjustment points. However, the MMS conducts surveys while moving along the crest of the levee; hence, in some cases, the laser is not emitted from the levee slope. Therefore, subsidence areas can be detected at the crest of a levee with high accuracy. However, there are no cases of practical application as a method for identifying areas that require countermeasures for the entire levee, including the slope. Three-dimensional data in areas that the MMS cannot measure can be obtained by running an MMS along the toe of the slope [19,20,21]. However, there is an issue with the limited number of areas in which the MMS can be run continuously. Trials of an MMS surveying system have been reported in an attempt to address this issue, in which the scanner is installed 3.5 m above the ground to reduce blind spots in the laser irradiation range [22].

**Figure 3:**
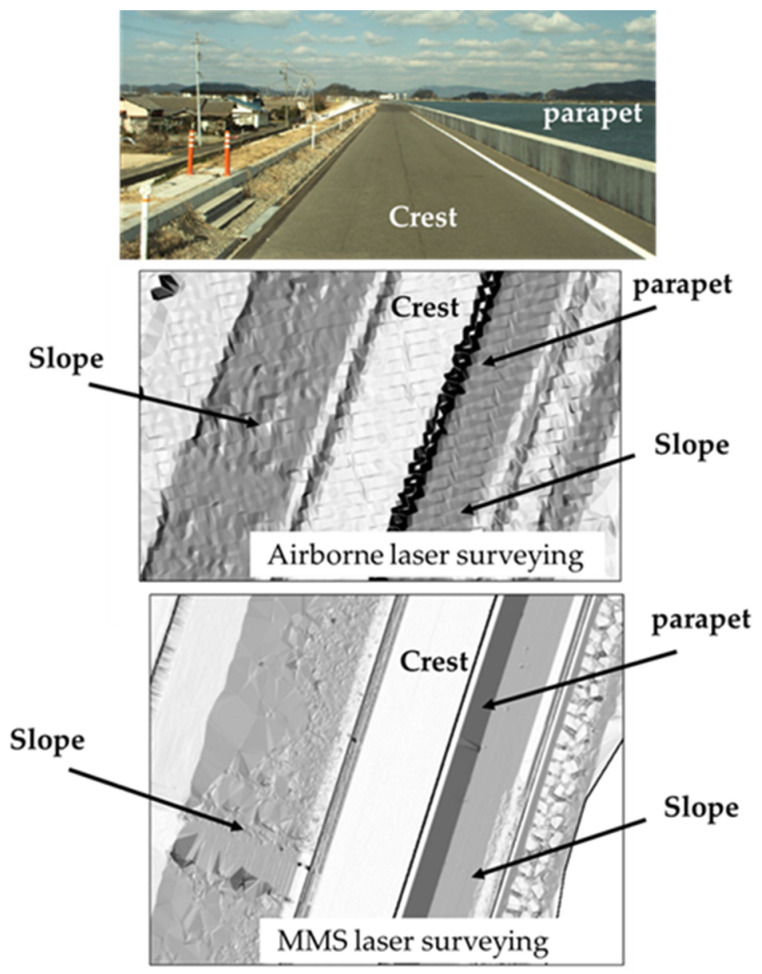
Comparison of results from airborne laser and MMS laser surveys.

**Figure 4:**
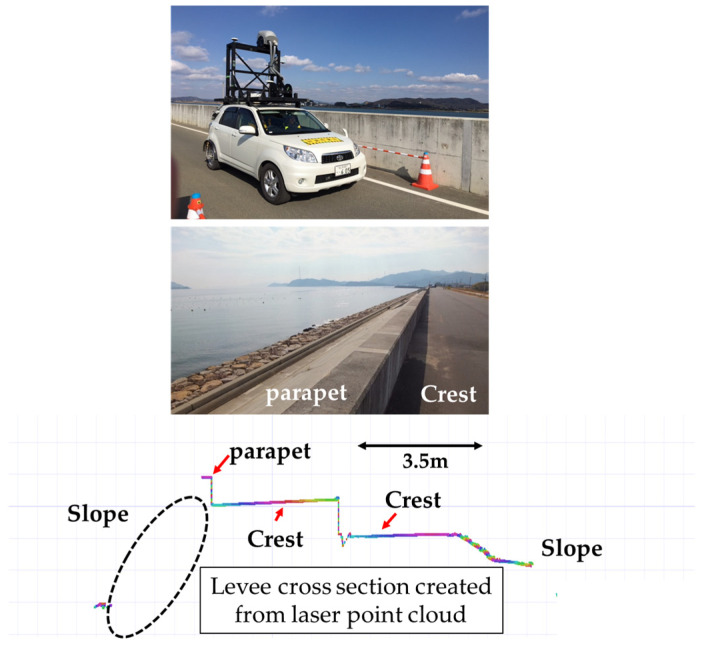
Laser point cloud distribution using MMS irradiated from a height of 3.5 m.

Figure 3 compares the results of airborne laser surveying with those of an MMS laser surveying system with a laser scanner installed 3.5 m above the ground. Commonly used airborne laser surveying achieves a laser point cloud spacing of at least 0.50 m, which causes the top of a recurved parapet wall, which is approximately 0.40 m wide, to appear wavy, as shown in the figure. This makes accurate reproduction of the target object with precise coordinate values impossible. By contrast, MMS surveying allows for high-density laser point cloud spacing, clearly reproducing the shape of the top of the recurved parapet wall. As such, the MMS has the advantage of enabling easy long-distance and high-precision surveying. The diagram of a levee represented with shading corresponding to point cloud density clearly shows that the riverside slope does not exhibit uniform shading, that is, a uniform point cloud density [23,24]. Figure 4 shows a detailed examination of the distribution of laser point clouds acquired by the MMS using laser irradiation from a height of 3.5 m. The figure is a cross-section, and the top of the recurved parapet wall can be surveyed. However, the laser point cloud on the slope surrounded by the dotted line is missing, indicating that the entire slope could not be surveyed. This is because the recurved parapet wall prevented the laser beam from reaching certain areas.

On this background, in the present study, we explore the challenges of MMS surveying, including when irradiating from a height of 3.5 m, and we discuss methods to resolve these challenges. Specifically, we explore the problems caused by blind spots in laser beams and discuss the advantages of acquiring 3D data to observe levee topography by irradiating the levee from higher elevations with lasers. Next, we use this discussion as a basis to report a case study in which noteworthy topographical features of levees were extracted from the perspective of assessing their integrity. River levees are large structures built on complex ground and have uneven geological structures. Technology for detecting subtle changes in levees can be used to assess their integrity even without directly accessing internal geological information, thereby enabling efficient river levee management [25,26,27].

## 2. Challenges of MMS Laser Surveying

In this section, we revisit the advantages and limitations of MMSs and discuss 3D data acquisition methods to address these challenges [28,29].

### 2.1. Verification of Characteristics of MMS Surveying System

MMS technology has been used to create road registers and investigate road surface conditions, leveraging its ability to survey large areas quickly and easily using vehicle traffic. As mentioned above, attempts have been made to install laser scanners at high altitudes to survey the topography of river levees. However, systems in which the surveying equipment is mounted on the top of a vehicle, as shown in Figure 2, are most commonly used. Herein, to examine the characteristics of laser surveying as the vehicle moves, we first discuss how the laser point cloud density and surveying accuracy are affected by the vehicle’s traveling speed and laser irradiation angle in a general-purpose MMS. The laser used in this study has a near-infrared wavelength, and when the object is wet, the laser’s reflection intensity decreases. In this study, the levee and vegetation were not wet, so the laser’s reflection intensity did not change depending on the object.

The laser scanners used in the experiment had a maximum irradiation distance of 500 m, a scan rate of 100 Hz, and a pulse rate of 300 kHz. To acquire a high-density laser point cloud, two scanners are installed as shown in Figure 2. They are installed at 45° angles to the left and right of the vehicle’s direction of travel, and the field of view, or the total area acquired by each scanner in one scan, is 360° vertically. The laser scanners were fixed in place on the top of the vehicle, and the laser irradiation position was at the same height as the vehicle. A characteristic of surveying using a laser scanner was that the laser point cloud density generally decreased with increasing irradiation distance. In this study, we investigated a method for assessing the soundness of a levee based on its topographical features and, therefore, first focused on laser irradiation density, which is related to the reproducibility of the levee’s shape. Specifically, we discussed how the decrease in laser point cloud density with laser irradiation distance is influenced by the vehicle’s traveling speed.

**Figure 5:**
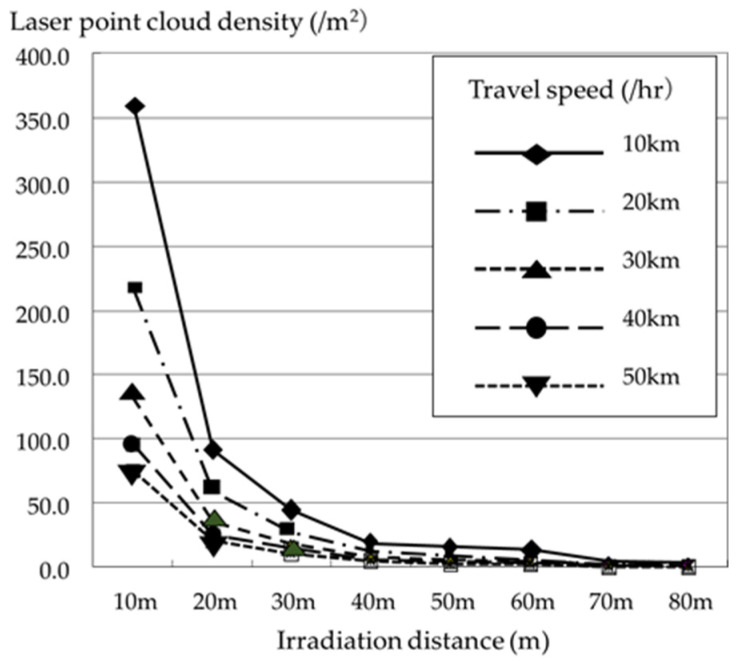
Relationship between laser point cloud density, travel speed, and irradiation distance.

Figure 5 shows the experimental results. Here, circular test specimens with a diameter of 1 m were placed at 10 m intervals, 10 to 80 m away from the vehicle’s traveling point. The laser was irradiated while the vehicle traveled back and forth at different traveling speeds, and the number of laser point clouds projected on the test specimen was defined as the laser point cloud density. The figure shows that the laser point cloud density is influenced by the distance from the vehicle and vehicle’s speed. We can confirm that this density decreases rapidly with distance from the vehicle, and as the vehicle speed increases, the laser point cloud density at the same irradiation distance decreases. This MMS specification used a laser scanner with a 500 m irradiation distance, but we can see that, at a driving speed of 20–30 km/h, which does not interfere with vehicle movement during surveying, a high-density laser point cloud can only be obtained within a range of about 20 m. Generally, the spacing of laser point clouds is determined by the vehicle’s driving speed, pulse rate, and scan rate. For example, when traveling at 20 km/h, with a scan rate of 100 Hz, that is, 100 scans per second, the spacing of laser points in the direction of travel is approximately 0.056 m. Furthermore, in the direction perpendicular to the direction of travel, with a pulse rate of 300 kHz and a scan rate of 100 Hz, the laser irradiation frequency will be 3000 times per cycle, and the laser will be emitted at intervals of approximately 0.12°, resulting in a laser point spacing of 0.021 m at a distance of 10 m. Increasing vehicle speed will result in increasing laser point spacing in the direction of travel. This is also affected by the divergence angle of the laser beam; hence, as shown in the experimental results, the laser point cloud density exhibits a considerable decrease with increasing irradiation distance and vehicle speed. Surveying using a moving vehicle is thus convenient; however, obtaining a uniform laser point cloud density for targets at both close and long distances with a general-purpose MMS is difficult.

Next, we examined the effect of the depression angle on the surveying accuracy when lasers are emitted from the top of a levee. When surveying from the top of a levee, the irradiation distance and incident angle of the laser beam differ at each point on the slope. The laser illumination area, known as the footprint, varies depending on the incidence angle of the laser beam, suggesting that surveying accuracy will not be uniform across the entire slope. Therefore, we used the results of MMS surveys conducted by traveling at the crest to examine surveying accuracy on slopes, with the depression angle as a parameter. Here, several circular test specimens with a diameter of 1 m were placed on the levee slope at various depression angles from the vehicle. The coordinates of their centers of gravity were calculated from the laser point cloud projected onto the test specimens. The position coordinates of the test specimen centers were then measured using network RTK single-point observation (VRS-GNSS surveying). These were considered the true values and compared with the coordinate values of the test specimens’ centers of gravity measured by the MMS. For each specimen, we calculated the coordinates of the center of gravity using the values measured by MMS and the coordinates of the center measured by VRS-GNSS surveying. In this study, the absolute value of the difference between these two coordinate values is defined as the surveying accuracy.

**Figure 6:**
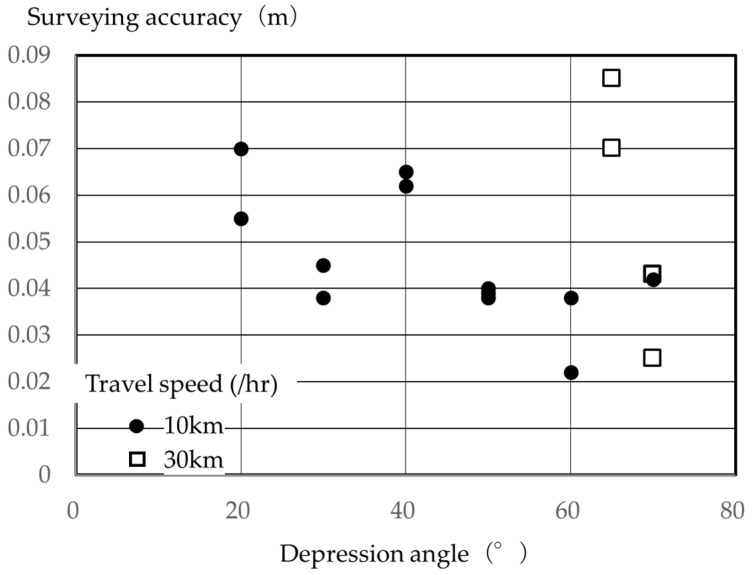
Relationship between depression angle and surveying accuracy (horizontal direction).

**Figure 7:**
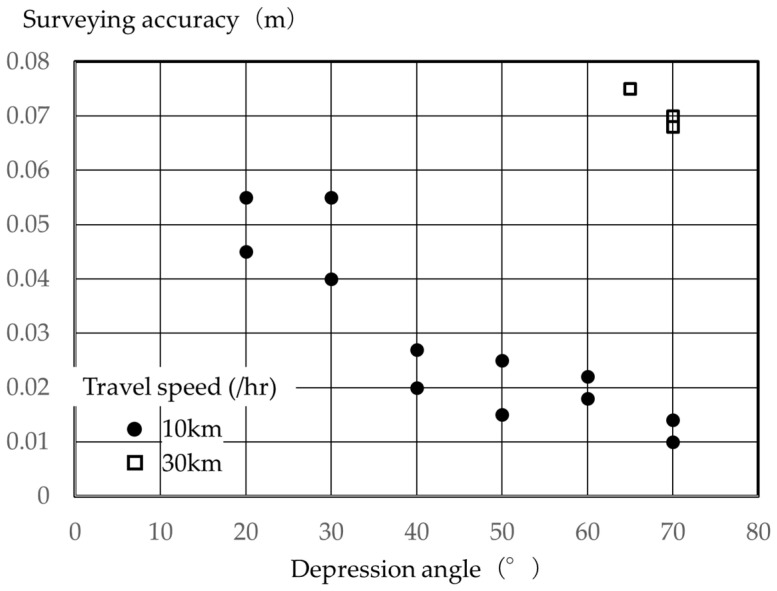
Relationship between depression angle and surveying accuracy (vertical direction).

Figure 6 shows the average difference between the coordinate values in two horizontal directions, and Figure 7 shows the difference between the coordinate values in the vertical direction. The figures show that the difference tended to increase with decreasing depression angle, both in the horizontal and vertical directions, with the difference becoming extremely large with increasing vehicle traveling speed. As seen in Figure 5, this can be attributed to the laser point cloud density decreasing with vehicle speed, making it impossible to ensure a sufficient number of laser points to accurately recognize the central coordinates of the verification points. The figure showed little survey data at 30 km/h, and no verification results at driving speeds greater than 30 km/h. This occurred because it became clear that surveying the entire slope with an accuracy of ±50 mm or less was difficult, and data could not be collected. As mentioned above, some method has been reported in which a laser scanner was installed at a height of 3.5 m and MMS surveying was performed while driving. However, considering that a standard levee cross-section with a gradient of approximately 26.6 degrees, the depression angle at the foot of the slope is 40° or less. Therefore, even in previous research, the results suggested that high-precision surveying is challenging with a uniform laser point cloud density all the way to the foot of the slope [30,31,32].

**Figure 8:**
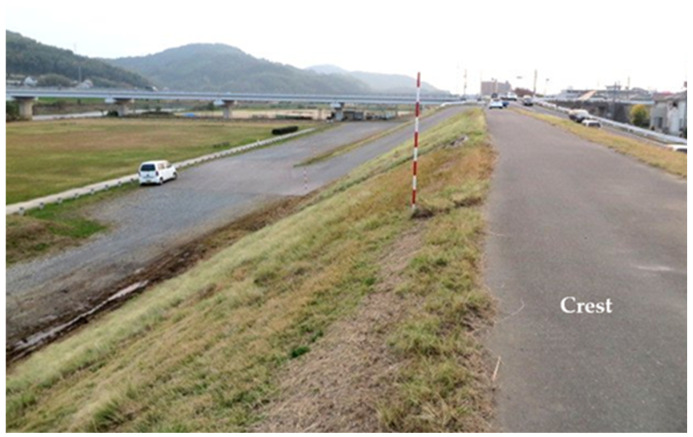
Vegetation growth on the levee slope.

**Figure 9:**
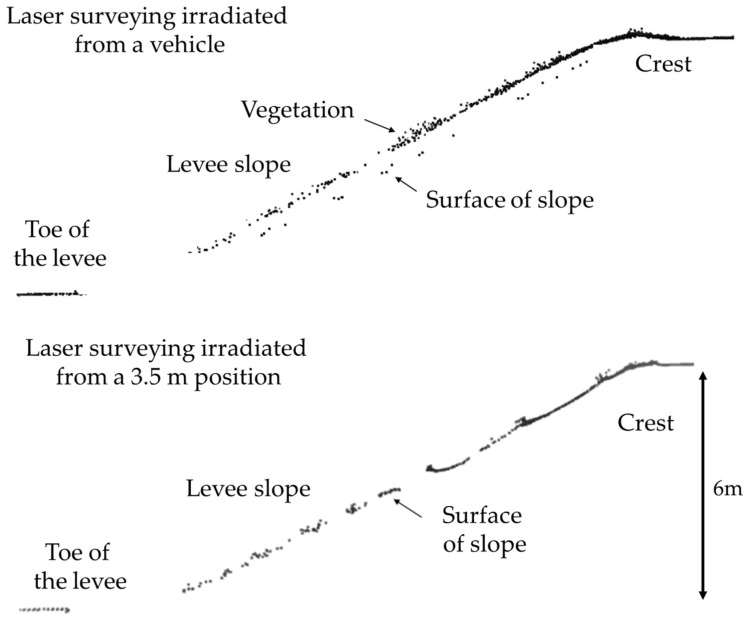
Point cloud distribution with different laser irradiation positions.

Furthermore, obtaining a uniform laser point cloud across the entire slope is often difficult on actual river levees due to obstacles such as vegetation and berms. Therefore, we investigated how the laser point cloud density, on a 6.0 m high levee with a gradient of approximately 26.6 degrees and dense vegetation, changes depending on the height of the irradiation position when a laser is emitted from the top. Figure 8 shows the on-site situation and Figure 9 shows a cross-section of the levee created from point clouds obtained by laser surveying irradiation from a vehicle, the ground, and an altitude of 3.5 m. As seen from the figure, when the laser beam was irradiating from a vehicle, less of the laser beam penetrated the vegetation, resulting in a low laser point cloud density on the ground surface. Furthermore, when the laser beam was irradiating from a 3.5 m position, it penetrated a higher proportion of the vegetation; however, several regions with a missing laser point cloud were observed on the slope. Neither of these locations allows us to observe the condition of the slope, including the region near the toe of the levee. Based on these results, we reconsider a method for 3D surveying of the entire levee.

### 2.2. Verification of Method That Enables Entire Levee to Be Surveyed

**Figure 10:**
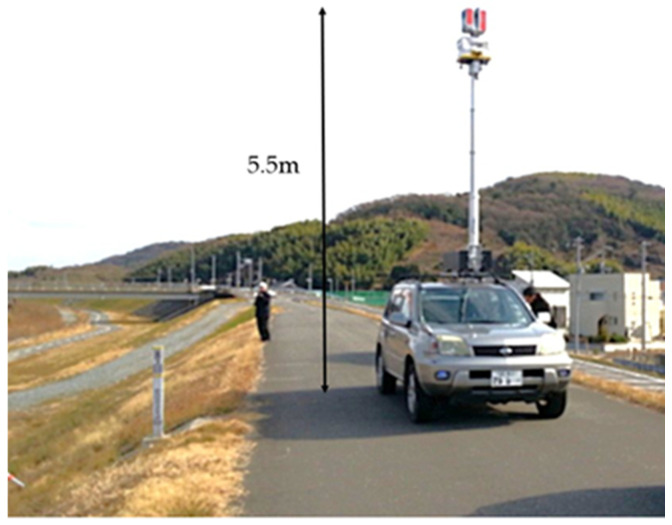
Setup of vehicle-mounted static laser scanning irradiating at a height of 5.5 m.

In the previous section, we discussed the challenges of surveying using a moving vehicle. To address this problem, we use a surveying system, as shown in Figure 10, that is equipped a laser scanner with a projection distance of approximately 190 m and a pulse rate of 1000 kHz and a scan rate of 200 Hz, installed 5.5 m above the ground. The stability of the mast on which the scanner is mounted has a significant impact on surveying accuracy. The mast is designed to be sturdy enough not to vibrate in wind speeds up to 5 m/s, and the scanner is equipped with an inertial measurement unit (IMU) consisting of an angular velocity sensor and an acceleration sensor. The IMU sensors detect the magnitude of the scanner’s translational and rotational motion, respectively, and use these measurements to confirm that surveying accuracy is not affected by the scanner’s attitude. Our proposed method can be called “vehicle-mounted static laser scanning”. We then evaluate and discuss its usefulness. The laser scanner is mounted on a vehicle to maintain the convenience of vehicle-based laser surveying. In terms of scanner performance, increasing the irradiation distance increases the scanner’s cost. The experiment in Section 2.1 revealed that even when the traveling speed was reduced, a scanner with an irradiation distance of 500 m could only obtain a high-density point cloud over a range of 20 m. The straight-line distance from the crest of the levee to the toe of the slope is approximately 14 m. Therefore, the irradiation distance of 200 m or less is considered acceptable. The pulse rate and scan rate are set to irradiate laser at a laser point spacing of 0.02 m, similar to the scanner used in the experiment in Section 2.1, at a distance of approximately 14 m.

**Figure 11:**
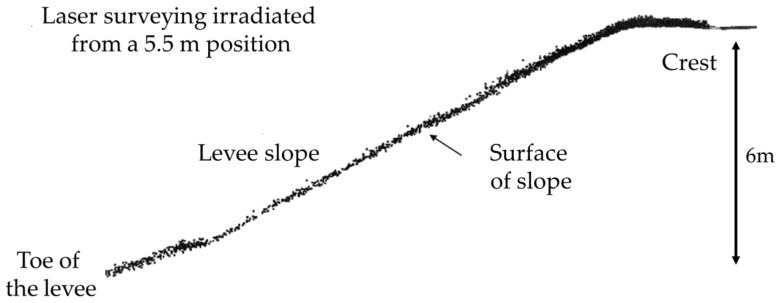
Cross-section of levee measured by laser irradiated from a height of 5.5 m.

**Figure 12:**
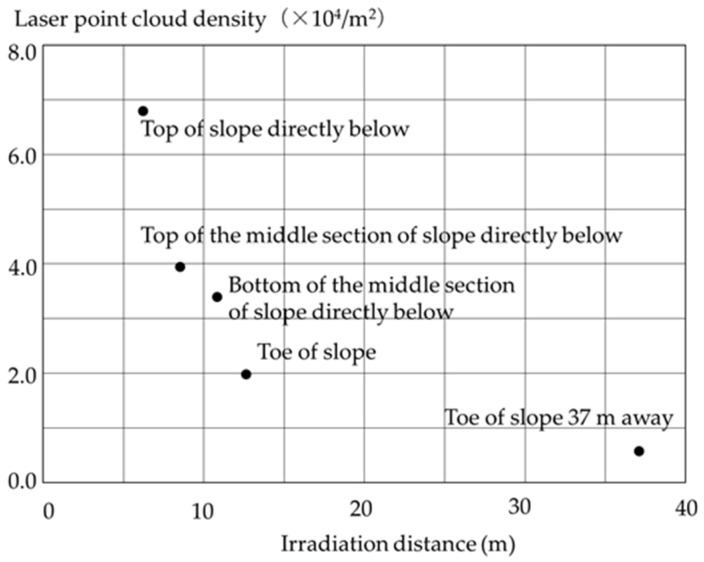
Laser point cloud density measured by laser irradiated from a height of 5.5 m.

Figure 11 shows a cross-section of the levee shown in Figure 8. Unlike the cross-section shown in Figure 9, when irradiating from a height of 5.5 m, the density of the laser beam reflected from the ground surface was high, highlighting the advantages of laser irradiation from a high altitude. In other words, by using this method, it was possible to survey not only the levee crest, but the entire slope including the toe, which was not possible with conventional MMS. A cross-sectional view of the entire levee, such as the one shown in Figure 11, can also be obtained using a tripod-mounted terrestrial laser scanner, but in that case, it is necessary to repeatedly move the equipment to the crest and high-water channel, set it up, and survey. This method has the advantage of being efficient, as it allows the entire levee to be surveyed simply by moving along the crest in a vehicle. Figure 12 shows the results of a detailed investigation of this laser point cloud density. The laser point cloud density at each point on the slope on the cross-section of the laser irradiation position was referred to as that “directly below”. Although the laser point cloud density decreased considerably with irradiation distance even when irradiating from a stationary position, a density of approximately 1000 points per square meter was obtained even at the toe of the slope, enabling reconstruction of the shape of the entire levee with high accuracy. Table 1 lists the surveying accuracy on each slope. Accuracy is expressed as the difference obtained using the same method as shown in Figure 6 and Figure 7. Emitting the laser from a height of 5.5 m enabled a survey of the entire slope directly below and that within a radius of approximately 40 m with an accuracy within ±20 mm, and within ±50 mm, respectively. The results in Table 1 meet the required measurement accuracy of 50 mm to quantify deformation during river levee inspections [4]. The difference was within ±20 mm at the verification point installed on the crown at an irradiation distance of 40 m, suggesting the influence of the depression angle at the toe of the slope at a long distance, as seen in Table 1.

For maintaining road structures and preventing traffic hazards, vehicles over 4.1 m in height are generally difficult to use on roads that are designated by road administrators as safe. Therefore, during actual surveying, the vehicle remained stationary while repeatedly irradiating the area with a laser from a high altitude. Furthermore, as shown in Figure 5, the method of surveying while the vehicle is moving is influenced by vehicle speed, so conducting the survey while the vehicle is stationary has the advantage of ensuring consistent accuracy for the entire levee. The high-altitude laser irradiation in this method enables surveying a wider area. Thus, the inspection work can be made more efficient even when stationary surveying is repeated while moving. The accuracy of airborne laser surveying depends in part on the aircraft’s flight altitude, with measurements typically tending to be approximately ±150 mm in the vertical direction and less accurate than that in the horizontal direction. The high-altitude laser irradiation in the proposed method enables the entire levee shape to be captured with greater accuracy than airborne laser surveying, even in areas with dense vegetation.

## 3. Examination and Discussion of Usefulness of Proposed Method

In this section, we present the results of a field study involving topographic surveying of a levee using laser irradiation from a height of 5.5 m. The site was the estuary section of the Asahi River, which flows through Okayama City, Japan. The levee was 6.0 m high with a gradient of approximately 26.6 degrees and composed of sandy soil with a fine fraction of 32.1%. The top of the levee was paved with asphalt, and grass less than 0.3 m high grew on the slope. The 2013 amendment to the River Act requires technical standards to inspect river management facilities such as levees before flood season and typhoon season. The proposed method was applied and examined in areas where visual observations of deformation had been previously confirmed during regular inspections [33,34,35].

Here, we also attempted to extract deformation locations based on the visualization techniques used previously in airborne laser surveying during data processing. Specifically, we introduced a method for visualizing laser point clouds by transparently combining a slope map, indicating changes in slope, with an altitude gradient map, which colors the elevation distribution. This method depicts the topography by displaying a colored altitude gradient map with 50% opacity on top of a slope map with 100% opacity. This also prevents the inversion of ridges and valleys, and combines the important topographical elements of “elevation” and “slope”, thereby enabling an intuitive determination of the relative positions of point clouds of interest in topographic interpretation, or the difference in elevation of the terrain.

**Figure 13:**
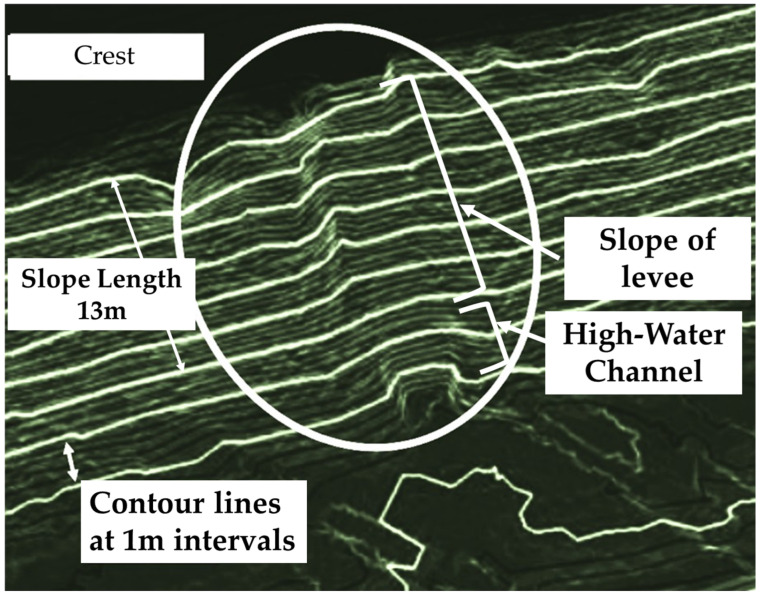
Elevation distribution measured by laser irradiated from height of 5.5 m.

A high-density laser point cloud of over 1000 points per square meter is used in Figure 13 to transparently combine an elevation cross-section map (a colored elevation map with 1.0 m intervals) with a slope map. It then draws contour lines to visualize the terrain in detail. The area surrounded by a solid line is the deformation location, and there are traces of gully erosion on the levee slope. Given that erosion is accelerated by rainwater, these changes must be monitored. The location of this deformation can be easily identified using the visualization technique shown in the figure. Subsequently, examining the laser point cloud of the discovered location in detail enables the quick and easy implementation of the process from measurement to quantification of the affected area. This in turn allows for the practical utilization of the characteristics of high-density 3D laser point clouds for detecting deformation.

**Figure 14:**
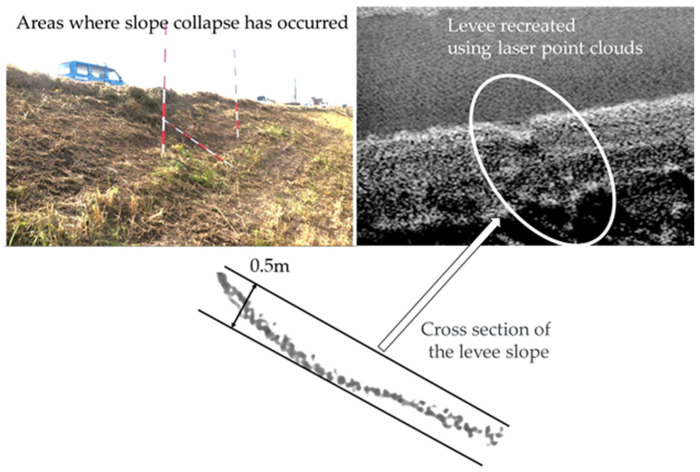
Deformation of levee slope detected by laser irradiated from height of 5.5 m.

Figure 14 shows the results of quantifying the deformation at the locations extracted in the diagram. In this study, we attempted to detect and quantify areas of slope collapse from a cross-section of the levee. A survey of the current location detected an undulation of approximately 0.5 m, and the same results were obtained using laser surveying. The levee slope is erosion-resistant because it is uniformly covered by vegetation; however, the presence of bare ground can indicate vulnerability. Even a small area of sparse vegetation or a depression on the slope can be a weak spot, allowing scouring to progress. Meanwhile, there have been cases reported where overflow occurred but the vegetation on the back slope prevented the levee from breaching. Furthermore, the shear force required for overflow water to destroy a levee is roughly proportional to the overflow depth. Any unevenness can lead to a proportional increase in depth. For example, if there is an unevenness of the same depth as a 100 mm overflow, then the overflow depth in that section will be doubled to 200 mm. Unevenness of a few dozen millimeters is not a major problem; however, unevenness of 100 mm or more can lead to the concentration of overflow water and increase the risk of levee breaches. Therefore, observations of the regions with sparse vegetation, of depressions, and of the progression of unevenness becomes important along with that of the slope collapses. Measuring large areas with high accuracy has been challenging so far, which in turn makes quantifying the progression of unevenness difficult. As discussed in the previous section, our proposed method can measure entire slopes over a wide area with a high accuracy within ±40 mm. This outcome enables even minute deformations to be quantified and areas requiring monitoring or countermeasures to be identified. Thus, by enabling high-precision surveying of the entire levee using high-density laser point clouds to detect deformations that may have been overlooked with the aforementioned MMS examples, our results suggest that this method can help overcome the limitations of MMSs.

## 4. Conclusions

In this paper, we discuss the challenges of vehicle-based laser surveying as a method for utilizing 3D data in river levee inspections, and summarize the outcomes of existing research to address these challenges. Specifically, existing vehicle-based laser surveying technology, which is being examined for use as an alternative to longitudinal surveying, appears to struggle in assessing the integrity of the entire slope due to blind spots caused by the slope gradient, as well as the influence of vegetation on the levee. In the present paper, we also discuss this aspect in relation to the depression angle and vehicle speed during laser surveying, and the laser surveying irradiation position that allows data to be obtained up to the foot of the slope. We then verified the surveying accuracy of a method where the laser irradiates from a height of 5.5 m. The results showed that abnormalities on slopes could be detected more effectively than with previous vehicle-based laser surveying while maintaining the efficiency of the surveying work. The frequent heavy rains because of climate change in recent years have resulted in an increasing tendency for serious damage caused by external water flooding, such as levee breaches. Additionally, the currently tightening financial situation for river management necessitates further improvements in the efficiency of conventional river management methods. Methods that use 3D levee data include the vehicle-based surveying discussed in this paper, as well as attempts to use airborne laser surveying and UAV-based laser surveying. Considering factors such as cost, surveying accuracy, and ease of work, the results suggest that the characteristics of each method can be utilized appropriately, and that surveying methods that leverage each method’s respective advantages can be useful in river management. In this context, the results of the present research demonstrate that terrestrial laser surveying, which is cheaper and easier to perform than UAVs, can be used as a method to improve work efficiency. The proposed method can be practical to use as a complement to the visual levee inspection method. In the future, we plan to further refine this technology by performing more verification in practical applications.

## Figures and Tables

**Table 1:** Measurement accuracy of laser irradiated from a height of 5.5 m.

Location	Difference (mm)
Top of slope directly below	10.9
Bottom of the middle section of slope directly below	13.6
Top of the middle section of slope directly below	12.9
Toe of slope	11.0
Toe of slope 37 m away	42.0

## Data Availability

The raw data supporting the conclusions of this article will be made available by the authors on request.
